# Comparison of Mucosal and Faecal Microbiomes in Patients With Cirrhosis

**DOI:** 10.1155/grp/6847983

**Published:** 2026-04-26

**Authors:** Caroline S. Stokes, Tatjana Türk, Frank Lammert, Beate Appenrodt

**Affiliations:** ^1^ Food and Health Research Group, Faculty of Life Sciences, Humboldt University Berlin, Berlin, Germany, hu-berlin.de; ^2^ Department of Medicine II, Saarland University Medical Center, Saarland University, Saarland, Germany, uni-saarland.de; ^3^ Health Sciences, Hannover Medical School (MHH), Hannover, Lower Saxony, Germany, mh-hannover.de; ^4^ Innere Medizin, St. Elisabeth-Krankenhaus, Köln, North Rhine Westphalia, Germany

**Keywords:** Child–Pugh, gut flora, liver disease, microbial, proximal intestine

## Abstract

**Objective:**

The colonic sigmoid mucosal microbiome is reportedly different from the faecal microbiome in patients with cirrhosis. This exploratory study is aimed at comparing the luminal and mucosal microbiome in patients with cirrhosis, with a specific focus on the proximal intestine.

**Methods:**

Mucosal and faecal samples were obtained from 12 patients with cirrhosis. The microbiome was quantified with V4 16S rRNA gene sequencing. Relative abundance, alpha and beta diversity were calculated, compared between the mucosal and faecal samples and correlated with stage of cirrhosis.

**Results:**

Faecal samples displayed lower microbial diversity than mucosal samples (Shannon diversity, p = 0.025) and the microbiome profiles differed significantly: Operational taxonomic units primarily of the phyla Firmicutes and Actinobacteria were more abundant in faecal samples, whereas biopsy samples contained units spanning all six phyla. Microbial composition of faecal samples were more similar to faecal samples from other patients rather than to the individual′s corresponding biopsy sample (principal coordinate analysis, p = 0.004). At the family level, Lachnospiraceae, Erysipelotrichaceae and Enterobacteriaceae were significantly more abundant in faecal samples, whereas biopsy samples contained more Streptococcaceae (p = 0.011) and Prevotellaceae (p = 0.031). Faecal samples from patients in Child–Pugh Stage C contained less Bacteroidetes but significantly more Streptococcaceae than Stage B samples (p = 0.04); however, biopsy samples did not differ significantly.

**Conclusions:**

This exploratory study in a small sample of patients with cirrhosis observed significant differences in the microbial signature of faecal versus biopsy samples from the proximal intestine. Future studies are needed to further investigate the relationship between different gastrointestinal microbial sites and cirrhosis.

## 1. Introduction

Liver cirrhosis involves the formation of scarred tissue (fibrosis), as a consequence of permanent liver damage and progressive loss of function [1]. Cirrhosis is mainly caused by a chronic infection with the hepatitis B or C virus, chronic alcohol consumption or metabolic dysfunction‐associated steatotic liver disease, and represents the end stage of chronic liver diseases [2, 3]. High mortality rates are associated with advanced cirrhosis, which often reflects the decompensated state [2, 4, 5], with complications such as ascites, hepatic encephalopathy (HE), jaundice, gastrointestinal bleeding or spontaneous bacterial peritonitis (SBP) [1, 4, 6].

Cirrhosis and its complications have been associated with the gut microbiome [7, 8], which is partly explained anatomically by the gut–liver axis, whereby the portal vein carries intestinal blood to the liver. Quantitative metagenomics identified 15 highly specific microbial genes distinguishing patients with cirrhosis from healthy controls [9]. Specifically, cirrhosis was associated with Enterobacteriacae predominance and reduced Lachnospiraceae and Ruminococcaceae [10, 11]. Gut dysbiosis with decreased microbial diversity is suggested to occur before cirrhosis development [12, 13].

The vast majority of clinical studies quantify the luminal microbiome (i.e., the microbiome contained in faeces) as a proxy for the gut microbiome and correlate these microbial signatures to disease endpoints. However, the luminal microbiome has been demonstrated to differ from the mucosal microbiome in healthy volunteers, and in patients with Clostridioides difficile infection [14, 15]. Moreover, Bajaj et al. [16] observed the colonic sigmoid mucosal microbiome to differ from the luminal microbiome in patients with cirrhosis, and changes in the former to influence HE, and highlighted the intestines as a site of bacterial translocation [16]. Changes in the mucosal microbiome appear to be specific to liver disease, since a recent study highlighted differences in rectal mucosal microbiota composition between healthy controls and patients with chronic liver diseases [13]. The reported discrepancies between the luminal and mucosal microbiomes and their associations specifically in patients with cirrhosis have not been fully defined, and the proximal intestine remains understudied. Herein, we compare the luminal versus mucosal microbiome in patients with cirrhosis, with a specific focus on the proximal intestine. We hypothesised that both sampling sites are characterised by distinct microbial signatures.

## 2. Patients and Methods

### 2.1. Patients

A total of 12 patients were recruited into this observation study at the Department of Internal Medicine II, Saarland University Medical Center. The following inclusion criteria determined eligibility to participate: laboratory and clinical or histological evidence of liver cirrhosis; age > 18 years; and current indication for diagnostic esophagogastroduodenoscopy (EGD) (within the setting of standard monitoring of cirrhosis). The following exclusion criteria precluded study participation: presence of diabetes mellitus Type 1 or 2; any malignant disease or HIV virus infection; chronic inflammatory bowel disease; occurrence of gastrointestinal bleeding; supplementation with probiotics; presence of active bacterial infections (e.g., an ascites aspirate with neutrophil granulocytes > 250/*μ*L, defining SBP; presence of a urinary tract infection (urinalysis with bacteria > 10^5^/mL), receiving a course of antibiotics; previous occurrence of SBP; hepatorenal syndrome Type I or II; transjugular portosystemic shunt; active drug abuse. Of note, the time restriction for the above exclusion criteria was based on < 28 days prior to study enrolment.

The study was performed according to the Declaration of Helsinki guidelines and was initiated following approval by the local Research Ethics Committee (Ärztekammer des Saarlandes; ref. 43/14). All patients provided informed consent before participation, after being fully informed about the study procedures involved.

### 2.2. Standard Biochemical Analyses

Blood sampling was obtained to measure the following parameters: liver function tests (LFTs) including, serum alanine aminotransferase (ALT), aspartate aminotransferase (AST), alkaline phosphatase (AP) and *γ*‐glutamyl transferase (*γ*‐GT) activities as well as serum bilirubin and albumin concentrations. In addition, triglycerides (TG), total cholesterol (TC), high‐density lipoprotein (HDL) cholesterol and low‐density lipoprotein (LDL) cholesterol were measured. Routine blood tests were conducted to quantify: sodium, potassium, creatinine, urea, glucose, creatinine kinase (CK), creatinine kinase‐muscle/brain (CK‐MB), C‐reactive protein (CRP), international normalised ratio (INR), partial thromboplastin time (PTT), *α*‐amylase, lactic acid dehydrogenase (LDH), cystatin‐C, N‐terminal pro B‐type natriuretic peptide (NT‐proBNP), 25‐hydroxyvitamin D, erythrocytes, haemoglobin, haematocrit, mean corpuscular haemoglobin (MCH), mean corpuscular volume (MCV), thrombocytes, leukocytes, lymphocytes, neutrophil granulocytes, eosinophil granulocytes, basophil granulocytes and monocytes.

### 2.3. Body Composition

Height was measured using the stadiometer seca217 (Seca, Hamburg, Germany) and the Body Composition Analyser mBCA515 (Seca) was used to determine body composition. The mBCA515 is based on bioelectrical impedance analysis (BIA), using multiple impedance frequencies of 5 and 50 kHz. It consists of an eight‐electrode segmental analysis and includes empirical linear regression models to evaluate fat‐free mass (FFM), fat mass (FM), total body water (TBW), intracellular water (ICW) and extracellular water (ECW).

### 2.4. Clinical Assessments

Patients underwent clinical assessments, including the completion of the Alcohol Use Identification Test (AUDIT) questionnaire to document alcohol consumption. Following the EASL Clinical Practice Guidelines [17], the diagnosis of alcoholic‐associated liver disease was suspected upon documentation of regular alcohol consumption of > 20 g/d in women and > 30 g/d in men together with the presence of clinical and/or biological abnormalities suggestive of liver injury; to exclude alternative or additional causes of liver injury further laboratory work‐up, including hepatitis B and C virus serology, autoimmune markers, transferrin saturation and alpha1‐antitrypsin was considered. Physical activity was captured using standard questions about frequency and intensity of activities undertaken. The characterisation and assessment of clinical severity of liver disease was carried out based on Child–Pugh stage and the Model for End‐Stage Liver Disease (MELD) score. The presence of ascites was confirmed based on clinical or available ultrasound findings. The critical flicker frequency (CFF) test (HEPAtonorm Analyser; Accelab, Kusterdingen, Germany) was used to assess HE, the details of which have been published previously [18].

#### 2.4.1. EGD

For the endoscopic assessment of varices, the endoscope was advanced beyond the Treitz band and inverted in the stomach after a 12‐h fast. Biopsies were taken with standard forceps from the proximal jejunum (1×) in the setting of routine biopsy sampling including Helicobacter pylori status. The biopsy samples were frozen immediately after removal at −80°C. In all patients undergoing EGD, the following coagulation parameters were documented: Quick > 50%, PTT < 50 s, platelets > 50G/L. All patients taking proton pump inhibitors paused treatment for at least 14 days before performing EGD.

#### 2.4.2. Stool Collection

Patients provided faecal samples during the clinic visit. The patients deposited the sample into four aliquots, which were immediately stored at −80°C.

### 2.5. Microbiome Analyses

Microbiome sequencing of the mucosal and faecal samples was conducted by Second Genome Inc. (San Francisco, California, United States) via V4 16S rRNA gene sequencing on the Illumina MiSeq platform, the full details of which are described in Supporting Information.

### 2.6. Statistical Analyses

The main study outcome was to determine whether differences exist between the luminal versus mucosal microbiome in patients with cirrhosis. All statistical analyses were performed using SPSS 24.0 (IBM, Ehningen, Germany) and GraphPad Prism 7.0 (GraphPad Software, California, United States). Given the small sample size, nonparametric statistics were used and descriptive data were reported with the median (minimum–maximum) or using frequencies. Within‐patient differences were tested with the Wilcoxon signed rank test, and differences between patients were evaluated with the Mann–Whitney U test. Alpha and beta diversity metrics were also used, in addition to ordination/clustering such as with principal coordinate analysis, sample classification, and whole microbiome or taxon significance testing. A two‐sided p value ≤ 0.05 was set as the threshold for statistical significance.

## 3. Results

### 3.1. Patient Characteristics

A total of 12 patients (75% were women) with cirrhosis were included in this observational study. Table 1 illustrates their clinical and biochemical characteristics. Two‐thirds of the patients were classed as having moderate cirrhosis, as reflected by Child–Pugh stage B. From the remaining patients, two each were categorised with Child–Pugh Stages A and C, respectively. Pre‐existing liver diseases included alcohol‐associated cirrhosis in five patients, chronic hepatitis C virus (HCV) infection in three patients and one patient suffered from both alcohol‐associated cirrhosis and HCV infection. Another patient had a diagnosis of chronic hepatitis B and D virus infection, one patient was diagnosed as having cirrhosis of genetic aetiology, and a single patient was diagnosed with cryptogenic cirrhosis.

**Table 1:** Clinical characteristics of the study cohort.

Sociodemographic characteristics	
N (men/women)	12 (3/9)
Age (years)	52 (31–62)
Liver disease and complications	
Child–Pugh Stage A/B/C	2/8/2
MELD score	12.5 (6.0–27.0)
Ascites	10
Varices	9
Hepatic encephalopathy	0
Medications	
Beta‐blockers, n (%)	7 (58)
Diuretics, n (%)	9 (75)
Lactulose, n (%)	6 (50)
Proton pump inhibitor, n (%)	8 (67)
Vitamins, n (%)	7 (58)
Body composition markers	
Body weight (kg)	69.1 (39.2–88.4)
BMI (kg/m^2^)	25.5 (16.5–34.5)
FFM (kg)	45.2 (30.7–52.7)
FM (kg)	26.6 (8.5–37.3)
TBW (kg)	33.0 (21.9–38.3)
Phase angle (°C)	4.0 (2.6–6.3)
Biochemistry	
Lipid profile	
Triglycerides (mg/dL)	85 (47–140)
Total cholesterol (mg/dL)	151 (80–351)
LDL cholesterol (mg/dL)	103 (58–297)
HDL cholesterol (mg/dL)	28 (10–126)
Liver profile	
AST, U/L	80 (63–200)
ALT, U/L	31 (17–229)
*γ*‐GT, U/L	128 (31–476)
AP, U/L	113 (85–221)
Albumin, g/L	36 (28–40)
Bilirubin, mg/dL	1.7 (0.8–7.4)
Clinical chemistry	
Sodium (mmol/L)	139 (133–145)
Potassium (mmol/L)	4.3 (3.6–4.6)
Creatinine (mg/dL)	0.98 (0.62–2.20)
Urea (mg/dL)	28.5 (13.0–54.0)
Cystatin‐C (mg/L)	1.6 (1.0–3.1)
Protein (g/L)	66.5 (62.0–90.0)
Glucose (mg/dL)	101 (84–143)
CK (U/L)	56.5 (20.0–363.0)
NT‐proBNP (pg/mL)	138.1 (45.4–1355.0)
CRP (mg/L)	7.5 (1.4–22.0)
INR	1.3 (0.9–1.9)
PTT (seconds)	30.5 (24.0–52.0)
LDH (U/L)	225 (190–366)
Amylase (U/L)	71 (46–177)
Blood count	
Erythrocytes (10^6^/*μ*L)	3.6 (2.5–4.7)
Haemoglobin (g/dL)	12.0 (7.8–14.2)
Haematocrit (%)	35.5 (24.0–40.0)
MCH (pg)	33 (23–39)
MCV (fl)	95 (73–112)
Thrombocytes (1000/*μ*L)	126 (46–350)
Leukocytes (1000/*μ*L)	5.9 (2.9–9.3)
Lymphocytes (%)	22 (6–42)
Neutrophil granulocytes (%)	62 (40–81)
Eosinophil granulocytes (%)	3 (1–8)
Basophil granulocytes (%)	1 (0–2)
Monocytes (%)	9 (7–17)

Abbreviations: ALT, alanine aminotransferase; AP, alkaline phosphatase; AST, aspartate aminotransferase; BMI, body mass index; CK, creatine kinase; CRP, C‐reactive protein; FFM, fat‐free mass; FM, fat mass; HDL, high‐density lipoprotein; INR, international normalised ratio; LDH, *α*‐amylase, lactic acid dehydrogenase; LDL, low‐density lipoprotein; MCH, mean corpuscular haemoglobin; MCV, mean corpuscular volume; MELD, Model for End‐Stage Liver Disease; NT‐proBNP, N‐terminal pro B‐type natriuretic peptide; PTT, partial thromboplastin time; TBW, total body water; *γ*‐GT, *γ*‐glutamyl transferase.

With regard to anthropometry, five patients had a BMI within the normal range (18.5–24.9 kg/m^2^), one patient was categorised as underweight, five patients as overweight (25.0–29.9 kg/m^2^) and one patient as obese. Phase angle, derived from BIA, is a reliable indicator of malnutrition [19]. A phase angle < 5.1° has been associated with an increased risk of death in patients with cirrhosis [20]. In this sample, only one patient had a phase angle > 5.1°, with the remaining 10 patients showing values considerably below this cut‐off (value was missing for one patient).

All patients reported following omnivore diets, and none used regular probiotic supplements. Six patients reported ≥ 2 bowel movements per day, whereas the remaining six reported one daily. Three patients were classified as drinking heavily (indicated by ≥ 8 points in the AUDIT questionnaire). Five patients were nonsmokers, one was a previous smoker and six patients were current smokers.

### 3.2. Differences in Microbiome Diversity Between the Luminal and Mucosal Samples

**Figure 1:**
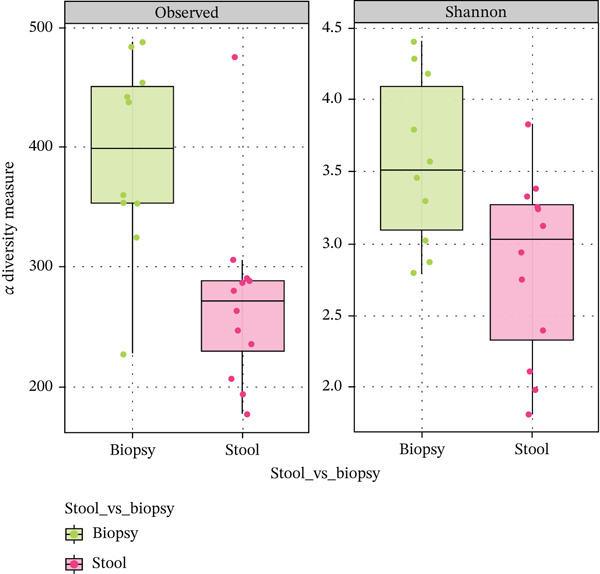
Alpha‐diversity estimates. (Left) Observed is the total number of operational taxonomic units (OTUs) present per sample, aka richness. (Right) Shannon refers to the Shannon Diversity Index, which accounts for both the abundance and evenness of OTUs present.

Faecal samples had a higher sequencing depth and lower richness when compared with biopsy samples. Thus, the diversity of faecal samples was significantly lower than that of the biopsy samples (Figure 1). This was assessed by two alpha diversity measures, that is, the observed sample richness reporting the total number of operational taxonomic units (OTUs) present per sample (stool: 271 ± 77 vs. biopsy: 393 ± 83; p = 0.025), and the Shannon diversity index, which accounts for the abundance and evenness of OTUs (stool: 2.84 ± 0.64 vs. biopsy: 3.57 ± 0.59; p = 0.025). Principal Coordinate analysis revealed that samples tended to cluster according to sample type and biomass (p = 0.004). In other words, faecal samples were more similar to faecal samples from other patients in the study rather than to the individual′s corresponding biopsy sample.

### 3.3. Luminal and Mucosal Samples Differed at the Phyla and Family Level

**Figure 2:**
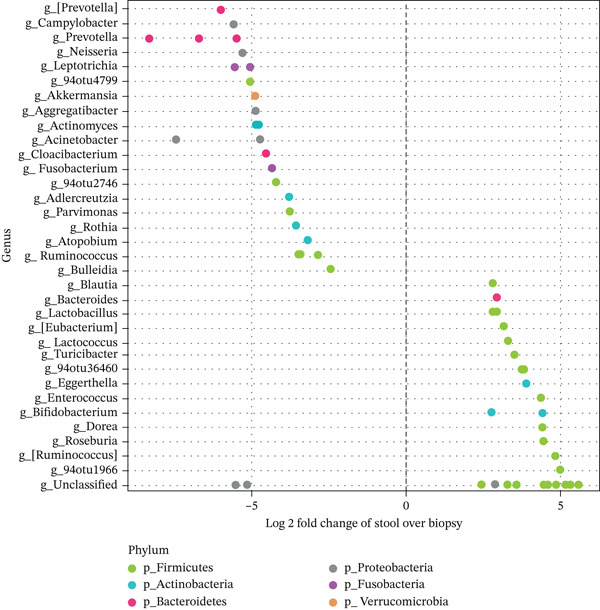
Differentially abundant features for sample pairs by subject ID. Each point represents an operational taxonomic unit (OTU) belonging to each Genus. Features were considered significant if their false discovery rate (FDR)‐corrected p value ≤ 0.05, and the absolute value of the Log‐2‐fold change was ≥ 1.

A total of 57 OTUs across several genera within six major phyla (Firmicutes, Actinobacteria, Bacteroidetes, Proteobacteria, Fusobacteria and Verrucomicorbia) were observed to differentiate faecal from biopsy samples. As illustrated in Figure 2, 27 OTUs, primarily of the phyla Firmicutes and Actinobacteria, were more abundant in faecal samples, whereas the 30 OTUs that were more abundant in biopsy samples spanned all six phyla. Of note, a particularly higher abundance of Prevotella sp. OTUs was present in biopsy samples.

**Figure 3:**
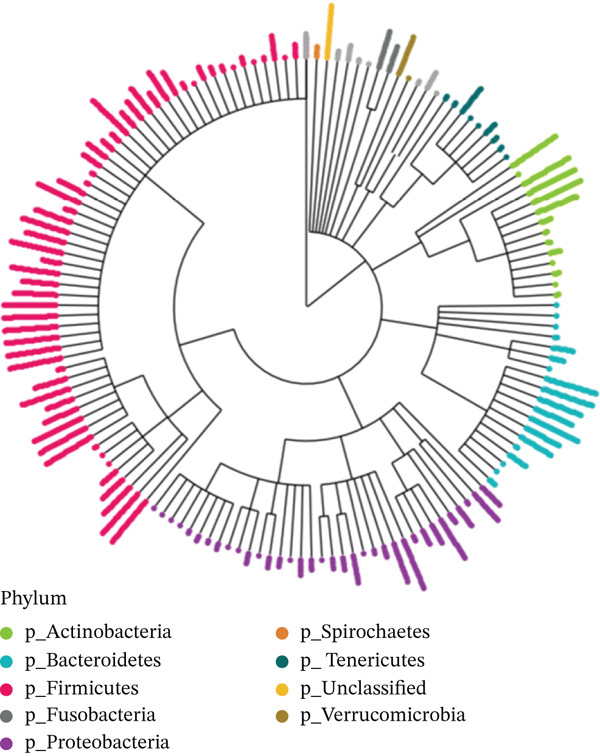
Phylogenetic tree at the Family rank. The height of each bar indicates the number of samples containing that particular Family. The most abundant Phylum‐level clades are coloured with the remainders in light grey.

**Figure 4:**
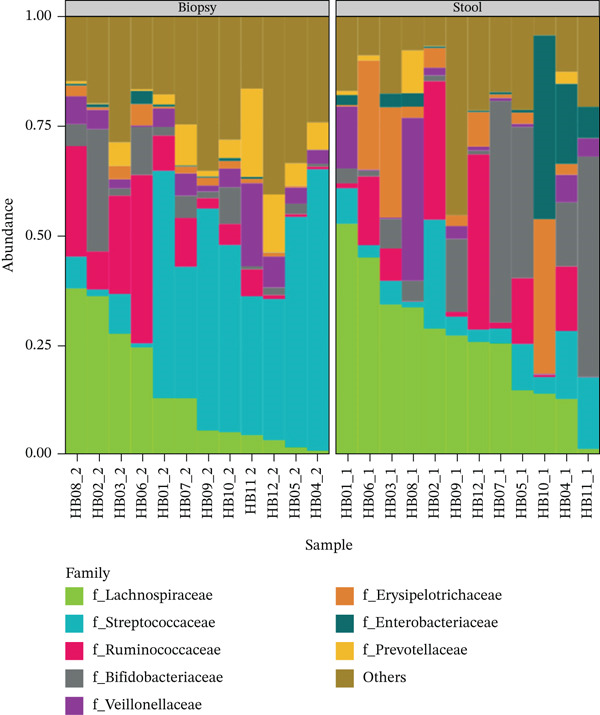
Proportional abundance. Plot shows the most abundant taxa at the Family level.

More than a quarter of the 142 families observed belonged to the phylum Firmicutes, as illustrated in Figure 3. The microbial community composition of the faecal and biopsy samples differed significantly from one another. At the family level, the following were significantly more abundant in faecal samples: Lachnospiraceae (26.2 ± 14.3 vs. 14.5 ± 13.6 in faecal and biopsy samples, respectively; p = 0.013), Erysipelotrichaceae (faecal: 9.06 ± 12.1 vs. biopsy: 1.55 ± 1.38; p = 0.025) and Enterobacteriaceae (6.47 ± 12.30 vs. 0.53 ± 0.81 in faecal and biopsy samples, respectively; p = 0.045), whereas Streptococcaceae and Prevotellaceae were significantly more abundant in biopsy samples (31.2 ± 22.0 vs. 8.29 ± 7.22; p = 0.011; and 5.67 ± 6.0 vs. 1.22 ± 2.8; p = 0.031 in biopsy and faecal samples, respectively). Figure 4 illustrates the most abundant taxa at the family level.

### 3.4. Differences in Luminal Samples at the Genus Level According to Stage of Cirrhosis

Faecal samples from patients in Child–Pugh Stages B and C differed significantly at the OTU and family level but no significant differences were observed in the proximal mucosal biopsy samples. Specifically, OTUs from the genus Bacteroidetes decreased in abundance relative to Child–Pugh class and were less abundant in patients with Child–Pugh class C. Moreover, the abundance of the family Streptococcaceae was significantly higher in the faecal samples of these patients (p = 0.04). However, the small sample size precluded further exploration of the novel finding of a lack of differences in proximal mucosal biopsies.

## 4. Discussion

Gut microbiome signatures hold potential to be used as biomarkers not only for liver disease detection but also to assess the risk of decompensation. Indeed, specific microbial signatures in this patient group have often been reported but the data are not yet consistent [21]. To add further to the inconsistencies, discrepancies between the luminal and mucosal microbiome have also been observed [16]. In this study, we hypothesised that the luminal and mucosal (proximal intestine) microbiome would be characterised by a distinct microbial signature in patients with cirrhosis, which was, to a certain extent, confirmed after 16S rRNA gene sequencing of mucosal and faecal samples from patients with cirrhosis.

Overall, the microbial diversity of the faecal samples from the patients in this study was lower when compared with mucosal samples. Differences between the microbiome composition of luminal (faecal) and mucosal samples (obtained via biopsy) have been previously shown in patients with cirrhosis [16]. For instance, Bajaj et al. [16] reported faecal samples to be enriched with Firmicutes (Lachnospiraceae, Veillonellaceae and Leuconostocaceae) in patients with cirrhosis, which are broadly consistent with the observations herein, where we found a higher abundance of Firmicutes (Lachnospiraceae) but also Erysipelotrichaceae and Enterobacteriaceae. The mucosa displayed a different abundance and included Firmicutes (Incertae Sedis XI and XIV), Actinobacteria (Propionibacteriaceae and Streptomycetaceae) and Proteobacteria (Vibrionaceae). A recent study observed patients with chronic liver disease to display an abundance of proinflammatory taxa (e.g., Proteobacteria, Enterobacteriaceae, Escherichia and Shigella), together with reduced alpha diversity in colonic mucosal samples as compared with healthy controls [13]. In contrast, our findings reported the dominant microbiome of the mucosa was Streptococcaceae and Prevotellaceae. The differences in mucosal microbiome abundance might be related to the different locations of mucosal biopsy, which included the rectosigmoid mucosa [16] and the rectal mucosa [13] in the studies above versus the proximal jejunum in our study.

The aetiology of chronic liver disease has previously been reported to play a critical role in determining the direction of microbiome alterations in patients with end‐stage liver disease [7, 12]. Enrichment in Prevotellaceae has been observed in patients with alcohol‐associated cirrhosis as compared with HBV‐related cirrhosis and controls [22]. These findings are consistent with the disease aetiology of two patients included in this study. However, it has also been suggested that the aetiology of liver disease is less important [23]. Indeed, studies have reported specific microbiota to be associated with cirrhosis (regardless of aetiology), which include increases in Proteobacteria, Enterococcaceae and Streptococacceae and decreases in Bacteroidetes, Ruminococcus and Lachnospiraceae [9–11, 22, 24]. Some of these findings were similar in our patients who displayed overall high levels of Enterococcaceae in faecal samples and Streptococacceae in mucosal samples. Moreover, Streptococcaceae has previously been associated with the severity of cirrhosis as determined by the Child–Pugh stage [22]. This finding was corroborated in our study with higher quantity of Streptococcaceae in patients in Child–Pugh Stage C as compared with patients in Stage B, whose faecal samples were predominantly characterised by the presence of Bacteroidetes.

A comparison of the microbiome across disease severity stages (e.g., Child–Pugh Stages B and C) revealed the faecal microbiota to differ, but not the microbiome of the proximal intestine biopsies. This finding suggests that dysbiosis in cirrhosis may be site‐specific. The mucosal microbiome has been suggested to be relatively more stable than the luminal microbiome. The latter is highly responsive to environmental factors such as diet and antibiotic use, whereas the mucosal microbiome is more strongly associated with host‐related factors such as immune function [25, 26]. Moreover, because both Child–Pugh stages B and C reflect advanced disease, and given the small sample size, potential differences might not be apparent and would need to be explored in a larger study.

Targeting the microbiome holds therapeutic potential for patients with liver diseases and although still in its infancy, it is suggested to influence the rate of decompensation [27], and numerous clinical benefits have been reported [28, 29]. Long‐term changes to both the mucosal and luminal microbiome were reported in patients with recurrent Clostridium difficile infection after receiving FMT [14]. Thus, FMT might hold potential to modulate upstream microbiomic signatures within the gastrointestinal tract [12, 30, 31]. The THEMATIC trial (a Phase 2 double‐blind placebo controlled randomised trial) in patients with cirrhosis, reported FMT as being safe, independent of dose, route or donor used [32]. They observed the placebo group to have the highest HE recurrence rate in a post hoc analysis. Conversely, this group also had lower baseline Lachnospiraceae.

The small sample size of this patient group is a limitation of this study, as is its exploratory design that precludes any causality inference. The reported findings are nevertheless broadly in line with other studies assessing the microbiome in patients with cirrhosis. Our results might also have been influenced by the heterogeneity of cirrhosis aetiologies. Additionally, antibiotic intake may affect the microbiome with long lasting impact [33, 34]; however, we did not systematically document data on previous antibiotic intake, which might have influenced microbiome profiles, particularly if taken frequently in the past. Thus, it remains unaccounted for as potential confounder. Moreover, the influence of current medication on the gut microbiome could not be explored; therefore, the possibility that drugs such as proton pump inhibitors might have altered the microbiota composition in patients with cirrhosis is acknowledged [35]. Over half of the patients in our study were taking lactulose; however, this has been reported to have no effects on the gut microbiome of patients with cirrhosis with regard to alpha and beta diversities, and bacterial taxa abundance [16, 36]. Moreover, the microbiome of the upper gastrointestinal tract differs substantially from that of the colon [37]. Given that we included biopsy data from the upper gastrointestinal tract, we were unable to fully compare our microbiome data with existing studies that have focused on colonic microbiome signatures. The strength of the current study, however, is the acquisition of not only faecal specimens but also endoscopically obtained biopsy samples from the upper gastrointestinal tract in all included patients.

In conclusion, the present exploratory study in a small sample of patients with cirrhosis observed marked differences in the microbial signatures of faecal versus biopsy samples from the proximal intestine, with the latter showing a greater degree of microbial diversity. Moreover, faecal and biopsy samples showed greater intersample similarity (to samples extracted from the same location), rather than to within patient similarity. Faecal but not mucosal microbiomes of patients differed based on the severity of cirrhosis, suggesting that dysbiosis may be site‐specific. Future biopsy‐based studies are needed to further investigate the relationship between different microbial sites within the gastrointestinal tract in this specific patient group.

## Author Contributions

C.S.S. and B.A. designed the study and analysed the clinical data. C.S.S. drafted the manuscript. All authors revised the manuscript for important intellectual content and approved the final version.

## Funding

This study was supported by Universitätsklinikum des Saarlandes HOMFOR (T201000736). Open Access funding enabled and organized by Projekt DEAL.

## Disclosure

All authors revised the manuscript for important intellectual content and approved the final version.

## Ethics Statement

All authors declare that the study was performed according to the guidelines set out in the Declaration of Helsinki and was initiated following approval by the local Research Ethics Committee (Ärztekammer des Saarlandes; ref. 43/14). All patients provided informed consent before participating in the study, after being fully informed about the procedures involved.

## Conflicts of Interest

The authors declare no conflicts of interest.

## Supporting information


**Supporting Information** Additional supporting information can be found online in the Supporting Information section. Full details of the microbiome sequencing of the mucosal and faecal samples as well as the statistical analyses are described in Supporting Information.

## Data Availability

The data that support the findings of this study are available on request from the corresponding authors. The microbiome based data are not publicly available due to the format of the data presentation as created by the bioinformatics outsourced company Second Genome but will be available upon request.
