# Supplementary material for: Comparison of Mucosal and Faecal Microbiomes in Patients With Cirrhosis
Source: Gastroenterol Res Pract. 2026 Apr 26;2026:6847983. doi: 10.1155/grp/6847983 (PMC13111803; doi:10.1155/grp/6847983)
Supplement: Supplementary file 1 — Supporting Information Additional supporting information can be found online in the Supporting Information section. Full details of the microbiome sequencing of the mucosal and faecal samples as well as the statistical analyses are described in Supporting Information. [file GRP-2026-6847983-s001.docx]

**Comparison of mucosal and fecal microbiomes in patients with cirrhosis**

Caroline S. Stokes^1,2^, Tatjana Türk^2^, Frank Lammert^2,3^, Beate Appenrodt^2,4^

^1^Food and Health Research Group, Faculty of Life Sciences, Humboldt University Berlin, 14195 Berlin, Germany

^3^Department of Medicine II, Saarland University Medical Center, Saarland University, Homburg, Germany

^3^Health Sciences, Hannover Medical School (MHH), Hannover, Germany

^4^Innere Medizin, St. Elisabeth-Krankenhaus, Köln

*Joint Correspondence: Prof. Dr. Caroline Stokes and Dr. Beate Appenrodt

Prof. Dr. Caroline Stokes

Food and Health Research Group, Faculty of Life Sciences, Humboldt University Berlin, 14195 Berlin, Germany Email: [caroline.stokes@hu-berlin.de](mailto:caroline.stokes@hu-berlin.de)

Dr. Beate Appenrodt

Department of Medicine II, Saarland University Medical Center, Saarland University

Kirrberger Str. 100, 66421, Homburg, Germany, [beate.appenrodt@hohenlind.de](mailto:beate.appenrodt@hohenlind.de)

**Supplementary Materials**

**Supplementary Materials: Extended Methods**

**Microbiome analysis**

Microbiome sequencing was carried out by Second Genome Inc. (San Franciso, CA) via V4 16S rRNA gene sequencing on the Illumina MiSeq platform. The procedure was carried out as follows, as documented by Second Genome:

**Sample Isolation:** Second Genome perfomed nucleic isolation with the MoBio PowerMag® Microbiome kit (Carlsbad, CA) according to manufacturer’s guidlines and optimised for high-throughput processing. All samples were quantified via the Qubit® Quant-iT dsDNA High Sensitivity Kit (Invitrogen, Life Technologies, Grand Island, NY) to ensure that they met minimum concentration and mass of DNA.

**Library Preparation:** To enrich the sample for bacterial 16S V4 rDNA region, DNA was amplified utilising fusion primers designed against the surrounding conserved regions which are tailed with sequences to incorporate Illumina (San Diego, CA) adapters and indexing barcodes. Each sample was PCR amplified with two differently bar coded V4 fusion primers. Samples that met the post-PCR quantification minimum and were advanced for pooling and sequencing. For each sample, amplified products were concentrated using a solid-phase reversible immobilisation method for the purification of PCR products and quantified by qPCR.

**Profiling Methods:** A pool containing 16S V4 enriched, amplified, barcoded samples were loaded into a MiSeq® reagent cartridge, and then onto the instrument along with the flow cell. After cluster formation on the MiSeq instrument, the amplicons were sequenced for 250 cycles with custom primers designed for paired-end sequencing. Samples are processed in a Good Laboratory Practices (GLP) compliant service laboratory running Quality Management Systems for sample and data tracking, detailed SOPs, regular audits, and quality control metrics are maintained for all sample handling, processing and storage procedures.

**Data Analysis**

The full data analysis included several distinct stages: pre-processing, summarisation, normalisation, alpha-diversity metrics (within sample diversity), betadiversity metrics, ordination/clustering, sample classification, and significance testing. Second Genome’s analysis software package was used for the microbiome-related statistical analysis.

**OTU Selection:** Sequenced paired-end reads were merged, quality filtered, and dereplicated with USEARCH (Edgar, 2013). Resulting unique sequences were then clustered at 97% similarity by UPARSE (de novo OTU clustering) and a representative consensus sequence per de novo OTU was determined. The clustering algorithm also performs chimera filtering to discard likely chimeric OTUs. Sequences that passed quality filtering were then mapped to set of representative consensus sequences to generate an OTU abundance table. Representative OTU sequences were assigned taxonomic classification via mothur’s bayesian classifier at 80% confidence; the classifier was trained against the Greengenes reference database of 16S rRNA gene sequences clustered at 99%. Taxa enclosed in brackets [] indicate proposed candidate names that have yet to be accepted by the International Journal of Systematic Bacteriology.

**Summarisation:** After the taxa are identified for inclusion in the analysis, the values used for each taxa-sample intersection are populated with the abundance of reads assigned to each OTU in an ‘OTU table’. A corresponding table of OTU Greengenes classification is generated as well.

**Alpha-diversity (within sample diversity) metrics:** ‘Observed’ diversity is the simply the sum of unique OTUs found in each sample, also know as sample richness. Chao1 calculates the estimated sample richness (number of OTUs) based on sequencing depth and taking into account rare taxa that may be present in a sample. Shannon diversity utilizes the richness of a sample along with the relative abundance of the present OTUs to calculate a diversity index.

**Beta-diversity (sample-to-sample dissimilarity) metrics:** All profiles are inter-compared in a pair-wise fashion to determine a dissimilarity score and store it in a distance dissimilarity matrix. Distance functions produce low dissimilarity scores when comparing similar samples. Abundance-weighted sample pair-wise differences were calculated using the Bray-Curtis dissimilarity. Bray-Curtis dissimilarity is calculated by the ratio of the summed absolute differences in counts to the sum of bundances in the two samples (Bray and Curtis 1957). The binary dissimilarity values were calculated with the Jaccard index. This metric compares the number of mismatches (OTUs present in one but absent in the other) in two samples relative to the number of OTUs present in at least one of the samples (Jaccard 1912).

**Ordination, Clustering, and Classification Methods:**

Two-dimensional ordinations and hierarchical clustering maps of the samples in the form of dendrograms were created to graphically summarize the inter-sample relationships. Principal Coordinate Analysis (PCoA) is a method of two-dimensional ordination plotting that is used to help visualize complex relationships between samples. PCoA uses the sample-to-sample dissimilarity values to position the points relative to each other by maximizing the linear correlation between the dissimilarity values and the plot distances. To create dendrograms, the samples from the distance matrix are clustered hierarchically using the ward method.

**Whole Microbiome Significance Testing:** Permutational Analysis of Variance (PERMANOVA) is utilized for finding significant differences among discrete categorical or continuous variables. In this randomization/Monte Carlo permutation test, the samples are randomly reassigned to the various sample categories, and the between-category differences are compared to the true between-category differences. PERMANOVA utilizes the sample-to-sample distance matrix directly, not a derived ordination or clustering outcome.

**Taxon Significance Testing:** Univariate differential abundance of OTUs is tested using a negative binomial noise model for the overdispersion and Poisson process intrinsic to this data, as implemented in the DESeq2 package (Love et. al. 2014), and described for microbiome applications in (McMurdie and Holmes 2014). It takes into account both technical and biological variability between experimental conditions. DESeq was run under default settings and q-values were calculated with the Benjamini-Hochberg procedure to correct p-values, controlling for false discovery rates.

**References**

Bray JR & Curtis JT (1957) An ordination of the upland forest communities of southern Wisconsin. Ecol. Monogr. 27:325–349

Edgar RC (2013) UPARSE: highly accurate OTU sequences from microbial amplicon reads. Nat Methods.10:996-998

Jaccard P (1912) The distribution of the flora in the alpine zone. New Phytologist. 11:37-50

Love MI, Huber W, Anders S (2014) Moderated estimation of fold change and dispersion for RNA-Seq data with DESeq2. bioRxiv. doi:10.1101/002832

McMurdie PJ & Holmes S (2014) Waste not, want not: why rarefying microbiome data is inadmissible. PLoS Computational Biology. 10: e1003531
